# Survey-based risk factors and genomic characterization of oral and oropharyngeal *Enterobacterales* carriage in humans and companion animals: a One Health study

**DOI:** 10.3389/fvets.2026.1900404

**Published:** 2026-09-21

**Authors:** W. Cooper Brookshire, K. Taylor Hellmann, D. Ashley Robinson, Keun Seok Seo

**Affiliations:** 1 Mississippi State University, Starkville, MS, United States; 2 The University of Mississippi Medical Center, Jackson, MS, United States

**Keywords:** antimicrobial resistance, *E. coli*, Enterobacterales, ESBL, *Klebsiella*, One Health, whole-genome sequencing, zoonotic risk

## Abstract

Antimicrobial-resistant (AMR) Enterobacterales, including extended-spectrum beta-lactamase (ESBL)- and carbapenemase-producing organisms, represent an important One Health concern involving humans, animals, and shared environments. This cross-sectional study evaluated survey-based risk factors for oral and oropharyngeal Enterobacterales carriage in humans and companion animals and characterized selected isolates using whole-genome sequencing (WGS). Human and companion animal samples were collected from participants presenting to the Mississippi State University College of Veterinary Medicine. A 27-item questionnaire assessed pet-related exposures, household antimicrobial use, occupational exposures, and human-animal interactions. Among 255 humans, 13.7% were positive for Enterobacterales and 7.5% for *Klebsiella* spp. Among 197 dogs, 47.2% were positive for Enterobacterales, including 30.5% positive for *E. coli* and 8.1% positive for *Klebsiella* spp. Only one of 18 cats was positive for Enterobacterales. In humans, veterinary occupational exposure demonstrated a nonsignificant positive trend toward *Klebsiella* spp. carriage (OR = 2.537, 95% CI: 0.963–6.688, *p* = 0.060). In dogs, antibiotic administration within the previous 12 months was associated with increased odds of *Klebsiella* spp. carriage (OR = 3.850, 95% CI: 1.046–14.173, *p* = 0.043) and *E. coli* carriage (OR = 3.049, 95% CI: 1.517–6.129, *p* = 0.002). Recent hospitalization within the previous 3 months was also associated with increased odds of canine *Klebsiella* spp. carriage (OR = 4.134, 95% CI: 1.400–12.213, *p* = 0.010). Whole-genome sequencing of 36 Enterobacterales isolates revealed substantial genomic diversity and identified multiple clinically important resistance determinants, including CTX-M-15 and NDM-5. A previously unassigned *Klebsiella pneumoniae* sequence type identified in both a human and canine isolate was subsequently assigned ST9790 through the Institut Pasteur Klebsiella MLST database. No shared bacterial species or sequence types were identified within matched human-animal pairs. These findings demonstrate associations between antimicrobial exposure and Enterobacterales carriage in companion animals and highlight the presence of clinically important antimicrobial resistance determinants in both humans and dogs. This study further supports the importance of continued One Health surveillance and antimicrobial stewardship efforts at the human-animal interface.

## Introduction

Antimicrobial resistance (AMR) remains one of the most critical public health challenges globally with resistant Enterobacterales, particularly extended-spectrum beta-lactamase (ESBL)-producing organisms and carbapenem-resistant Enterobacterales (CRE), identified as urgent threats by the Centers for Disease Control and Prevention (CDC) (1). Within the Enterobacterales order, *Klebsiella* spp. and others are significant pathogens responsible for severe community- and hospital-acquired infections in humans and companion animals (2, 3). Human infections caused by resistant *K. pneumoniae* and related species often result in substantial morbidity, mortality, and healthcare costs, with nearly half of CRE bloodstream infections proving fatal (4–6).

Companion animals, particularly dogs and cats, (herein, pets) increasingly share living spaces with their human owners, often in intimate proximity, creating potential pathways for interspecies bacterial transmission. Approximately 70% of U. S. households currently own pets, with dogs present in nearly half of these homes (7). These animals can harbor and transmit antibiotic-resistant organisms, serving as potential reservoirs or vectors for zoonotic pathogens, thus posing significant risks to public health (8, 9). Although the zoonotic potential of pathogens such as *Salmonella* spp., *Campylobacter* spp., and *E. coli* has been studied, the epidemiology of multidrug-resistant Enterobacterales transmission between pets and their owners remains underexplored, especially regarding *Klebsiella* spp. (9–11).

International data demonstrate that resistant Enterobacterales carriage is not limited to the United States. A global meta-analysis estimated that 16.5% of humans in community settings had intestinal carriage of extended-spectrum beta-lactamase-producing *E. coli*, with substantial regional variation (12). A separate global meta-analysis estimated prevalences of extended-spectrum beta-lactamase-producing *E. coli* of 6.87% in dogs and 5.04% in cats, based predominantly on fecal or rectal sampling and with marked geographic heterogeneity (13).

Recent reports highlight evidence of resistant bacteria, including ESBL and CRE organisms, in veterinary clinical settings (14, 15). Veterinary hospitals share several epidemiological risk factors with human healthcare facilities, such as widespread antimicrobial use, prolonged hospitalization, and indwelling medical devices, all of which are associated with heightened selection pressure and dissemination of resistant organisms (16–19). Clonal transmission of resistant Enterobacterales among animals within veterinary practices and boarding facilities further underscores these environments’ potential role in AMR epidemiology (20, 21).

To address this challenge, the National Institutes of Health (NIH) and CDC emphasize a “One Health” approach, advocating collaborative efforts between human, veterinary, and environmental health sectors to better understand AMR transmission dynamics and inform targeted intervention strategies (22, 23). Nonetheless, comprehensive surveillance data on AMR transmission pathways and associated risk factors between pets and their owners remain limited, particularly in community settings outside hospital environments. A recent clinical case at Mississippi State University College of Veterinary Medicine (MSU-CVM) documented CRE *K. pneumoniae* in a canine patient experiencing a severe urinary tract infection, with a closely related CRE isolate recovered from the dog’s oropharyngeal surveillance sample, prompting an investigation of bacterial carriage and transmission risks within pet-owning households in this geographic region (24).

Given this context, our study employed a combined epidemiological and genomic approach to investigate household-level risk factors for oral and oropharyngeal carriage of Enterobacterales in pets and their human owners. The specific aims were: (1) to identify survey-based risk factors associated with oral and oropharyngeal carriage of Enterobacterales, particularly *Klebsiella* spp., in pets and their owners; and (2) to characterize genomic features, resistance profiles, and sequence-type diversity among isolates recovered from human-pet pairs, thereby assessing the likelihood and extent of bacterial sharing at the household level. The findings of this research provide critical insights into potential zoonotic transmission pathways and inform evidence-based interventions to mitigate antimicrobial resistance risks within a One Health framework.

## Methods

### Survey design and implementation

A structured, cross-sectional questionnaire was developed to identify potential risk factors for the carriage of antimicrobial-resistant (AMR) Enterobacterales in pets and their owners. The questionnaire design drew upon established literature and prior surveys exploring human-animal interactions, antimicrobial use patterns, healthcare exposures, and environmental factors associated with AMR carriage (25, 26). It underwent expert review by infectious disease specialists, veterinary clinicians, and epidemiologists, followed by pilot testing with a subset of veterinary clinic clients to ensure clarity, comprehensibility, and relevance. The finalized questionnaire comprised 27 items categorized into three primary domains:

*Pet-related exposures*: Questions assessed antimicrobial use in pets within the past 3 and 12 months, veterinary hospitalizations, kenneling or boarding events, dietary habits (including raw meat consumption), and environmental behaviors such as drinking from toilets or natural water sources, and ingestion of fecal material.

*Owner and household exposures*: Questions assessed recent antimicrobial use and hospitalization events within the household (including both owners and co-inhabitants), occupational exposure risks related to employment in human or veterinary healthcare sectors, and international travel within the preceding year.

*Human-animal interface behaviors*: Questions assessed specific behaviors indicative of close physical contact between humans and pets, including frequency of owners kissing pets, pets licking owners’ hands or faces, pets’ sleeping arrangements (e.g., sharing a bed), and indoor defecation practices (e.g., litter boxes, pads).

All survey responses were formatted predominantly as categorical or binary options (e.g., “Yes,” “No,” or “Not sure”). Prior to statistical analysis, responses indicating uncertainty (“Not sure”) were grouped with negative responses (“No”) to conservatively estimate risk.

### Participant enrollment and survey administration

The survey was conducted from March to June 2023 at the MSU-CVM Animal Health Center. Human-pet pairs attending routine or specialty veterinary appointments were recruited using convenience sampling. Inclusion criteria required participants to be adults (≥18 years of age), capable of providing informed consent for themselves and their pets, and willing to participate in all aspects of the study, including the questionnaire and oral sample collection. Exclusion criteria included animals that could not safely undergo oral sampling. Human participants were also excluded if they indicated they had blood in their mouth, due to biosafety limitations of blood contaminated saliva sample handling. Informed consent was obtained from each participating pet owner, detailing study objectives, procedures, confidentiality measures, potential risks, and benefits. The study was approved by the Institutional Review Board (IRB-22-104) and Institutional Animal Care and Use Committee (IACUC-22-103). Once consented, participants were provided with a paper-based survey to complete at the time of enrollment. Completed surveys were securely stored, with electronic data managed in compliance with institutional data privacy and protection policies. Each human-pet pair was assigned a unique identifier to ensure confidentiality and allow linkage between survey responses and microbiological data. Enrollment was not restricted to a single one-human–one-pet dyad: multiple eligible adults could enroll in association with the same pet, and one adult could enroll with multiple pets.

### Statistical analysis of survey data

Descriptive statistics were computed for all survey items, providing frequencies and proportions of affirmative responses (“Yes”). For binary survey items, 95% confidence intervals (CIs) were calculated using exact binomial methods (Clopper-Pearson) to accurately estimate proportions. Bootstrap resampling methods (10,000 iterations) were utilized to derive 95% CIs for multi-category variables subsequently collapsed into binary outcomes for simplified analysis. Survey variables identified as potential risk factors for bacterial carriage outcomes were further analyzed using univariate logistic regression, conducted in IBM SPSS Statistics Version 29.0.2.0 (IBM Corp., Armonk, NY). Univariate logistic regression was performed to explore associations between survey variables and bacterial carriage outcomes. Regression sample sizes varied slightly by variable because of incomplete survey responses. Due to sparse outcomes and instability of multivariable models, including wide confidence intervals and non-convergence, results are limited to unadjusted odds ratios (ORs). To focus the primary manuscript tables on biologically plausible positive risk signals, variables were included in the main regression tables if OR > 1.0 and *p* ≤ 0.30. Complete univariate regression outputs are provided in Supplementary Tables 1–5.

### Sample size calculation

Based on published estimates, we assumed a *Klebsiella* spp. oral/oropharyngeal prevalence of approximately 10% in each group (27–29). A total of 200 human–pet pairs was considered a reasonable target to provide descriptive statistics for survey responses and prevalence estimates with acceptable precision (95% CI ± 4.2% for a 10% prevalence). This sample size also offers adequate power for exploratory risk-factor analyses, while yielding sufficient numbers of positive isolates to characterize genomic features and assess putative sharing between humans and their pets.

### Bacterial sample collection and handling

Oral rinse samples were collected from human participants, whereas oral/oropharyngeal swabs were collected from enrolled pets to assess carriage of Enterobacterales. Oral and oropharyngeal sampling approaches were selected because of ease of sampling and published reports demonstrating reasonably high Enterobacterales carriage rates, including Klebsiella spp., for both pets and humans (27–29). For pets, samples were obtained using sterile swabs (Starswab II, Starplex Scientific Corp, Etobicoke, Canada), which were gently swabbed along the buccal mucosa and the tonsillar crypt region by trained veterinary personnel. Swabs were then immediately placed into transport tubes containing modified Amies transport medium and stored under refrigeration (4 °C) until processing. For humans, samples were obtained using a sterile saline oral rinse technique. Participants were instructed to swish 20 mL of sterile, preservative-free saline (0.9% sodium chloride) within the oral cavity for 30 s. After swishing, participants expectorated the saline into sterile, labeled containers, which were then promptly refrigerated (4 °C) until microbiological analysis. All samples were processed within 24 h of collection to maintain organism viability and integrity.

The different sampling methods reflected feasibility and participant acceptability. Preliminary feedback indicated that veterinary clients were more willing to provide an oral rinse than undergo deep oropharyngeal swabbing. Because companion animals cannot perform an oral rinse, swabbing permitted trained personnel to target the buccal mucosa and tonsillar crypt region. Oral-rinse methods have previously been used to recover oral Enterobacterales from people (30).

### Sample processing and isolation procedures

Human oral rinse samples were homogenized by vortexing, and a 14-mL aliquot from each sample was centrifuged at 2,000 RPM for 7 min at room temperature. Then, the supernatant was poured off, leaving an estimated residual volume of approximately 0.5 mL. The bacterial pellet was resuspended in this residual saline to concentrate organisms before enrichment. The concentrated suspension was then inoculated into 2.5 mL of tryptic soy broth (TSB, Thermo Scientific, Waltham, MA) for enrichment. Pet swabs were similarly processed: each swab was vortexed in 2.5 mL TSB enrichment broth to dislodge organisms. All enrichment broths (both human and animal) were incubated aerobically in a shaking incubator at 37 °C for 4 h to facilitate bacterial recovery and proliferation. Following incubation, enriched broths were aliquoted (75 μL) into 3.6 mL of MacConkey broth and incubated aerobically overnight (18–24 h) at 37 °C and evaluated for color change or turbidity indicative of bacterial growth. The 75-μL inoculum into MacConkey broth was based on an established laboratory protocol and was selected to provide sufficient inoculum for bacterial recovery while limiting dilution of the selective broth and nonspecific turbidity caused by transfer of a larger enrichment-broth volume. If growth was not visible in MacConkey broths after 18–24 h, they were further incubated for an additional 24 h. All broths with color changes and/or turbidity were subsequently streaked onto MacConkey agar plates using a sterile 10 μL loop for isolation and phenotypic characterization. MacConkey agar plates were incubated aerobically overnight at 37 °C, and colonies exhibiting typical morphology consistent with lactose-fermenting Enterobacterales were selected for further identification/characterization.

### Bacterial identification

Isolated lactose-fermenting colonies were identified to the species level using the VITEK MS MALDI-TOF mass spectrometry system (bioMérieux, Durham, NC) following manufacturer instructions. Briefly, colonies were spotted onto disposable MALDI target slides and overlaid with matrix solution, then analyzed using MALDI-TOF mass spectrometry. Species-level identifications were accepted based on confidence values provided by the VITEK MS system. For statistical analyses and reporting, “any Enterobacterales” is defined as an isolate with lactose fermentation on MacConkey agar and species-specific analyses are based on Vitek MS speciation.

### Whole-genome sequencing (WGS) and genomic analysis

A subset of *Klebsiella* spp. and *Enterobacter* spp. isolates from the study were selected for further genetic characterization via whole-genome sequencing (WGS). The subset was selected based on isolate availability at the time of sequencing. DNA extraction was conducted using a commercial genomic DNA extraction kit (DNeasy Blood & Tissue Kit, Qiagen, Germantown, MD) following the manufacturer’s protocol. DNA quality and concentration were determined by spectrophotometry (NanoDrop, Thermo Scientific) and fluorometric quantitation (Qubit HS Assay Kit, Invitrogen). Genomic libraries were prepared using Nextera XT DNA Library Prep Kits (Illumina, San Diego, CA) following standard protocols. Sequencing was performed at the University of Mississippi Medical Center Molecular and Genomics Core Facility using an Illumina NextSeq 1,000 instrument with paired-end 150-bp read chemistry. Sequencing data were demultiplexed and adapter-trimmed using Illumina software. Further quality filtering included trimming low-quality bases (Phred quality score <Q13) and short reads (length <15 bp) using Trimmomatic v0.39 software. Sequences were assembled using Shovill v1.1.0 for *de novo* assembly and assessed for genome completeness and contamination using CheckM v1.2.3 (31–33). Genomes with <90% completeness and/or >10% contamination were excluded from analysis. Multilocus sequence typing (MLST) was performed using MLST v2.23.0 and resistance genes were identified using Abricate v1.0.1 and the Comprehensive Antibiotic Resistance Database (CARD) (34–37).

A focused Mash distance analysis and cgMLST assignment was performed for isolates sharing the same sequence type across host species. Mash distance was calculated using Mash v2.3 and 10,000 21-mers (38). cgMLST assignment was made according to their 629-loci (39).

## Results

### Prevalence of Enterobacterales

Microbiological analyses to determine oral and oropharyngeal carriage of Enterobacterales were performed on samples from 255 humans, 197 dogs, and 18 cats (Table 1). The discrepancy in sample numbers between humans and animals was due to difficulties in obtaining animal samples and cases where multiple human participants were linked to a single pet, or vice versa. Data are grouped and analyzed based on species (human vs. dog vs. cat) or human-pet pairs, where applicable. Among the 255 human samples, 35 (13.7%) were culture-positive for Enterobacterales, including 19 (7.5%) for *Klebsiella* spp., 6 (2.4%) for *Enterobacter* spp., and 1 (0.4%) for *E. coli*. Of the 197 canine samples, 93 (47.2%) were culture-positive for Enterobacterales, with *E. coli* most common (60/197, 30.5%), followed by *Klebsiella* spp. (16/197, 8.1%) and *Enterobacter* spp. (11/197, 5.6%). Of the 18 feline samples, only 1 feline sample (5.6%) was positive for Enterobacterales, precluding further analysis. Enterobacterales carriage was significantly higher in dogs (47.2%) than humans (13.7%, *p* < 0.001). *E. coli* carriage was more frequent in dogs (30.5%) than humans (0.4%, *p* < 0.001), while *Klebsiella* spp. prevalence was similar (humans: 7.5%, dogs: 8.1%). No human-pet household pairs shared matching bacterial species as identified by VITEK MS MALDI-TOF.

**Table 1:** Oral and oropharyngeal carriage of Enterobacterales.

Host	Total samples	Any Enterobacterales, *n* (%)	*E. coli*, *n* (%)	*Klebsiella* spp., *n* (%)	*Enterobacter* spp., *n* (%)
Human	255	35 (13.7)	1 (0.4)	19 (7.5)	6 (2.4)
Canine	197	93 (47.2)	60 (30.5)	16 (8.1)	11 (5.6)
Feline	18	1 (5.6)	0 (0)	0 (0)	0 (0)

### Descriptive survey results

A total of 221 surveys were completed, covering pet-related exposures, owner/household exposures, and human-animal interactions (Table 2). Nearly half of the pets (49.8, 95% CI: 43.0–56.6) received antibiotics in the past 12 months, and 38.5% (95% CI: 32.0–45.2) in the past 3 months. Pet hospitalization in the past 12 months occurred in 27.1% of pets (95% CI: 21.4–33.5). Owner antibiotic use in the past 12 months was reported by 43.0% (95% CI: 36.4–49.8), and 27.6% in the past 3 months (95% CI: 21.8–34.0) of respondents. Veterinary field employment was reported by 23.1% (95% CI: 17.7–29.2) of respondents. Owners frequently reported close contact: 71.0% regularly kissed their pets (95% CI: 64.6–76.9), 57.5% reported face-licking (95% CI: 50.7–64.1), and 78.3% hand-licking (95% CI: 72.3–83.5). Pets slept in owners’ beds in 52.5% of cases (95% CI: 45.5–59.7), and 18.6% regularly defecated indoors (95% CI: 13.7–24.3).

**Table 2:** Survey results.

Survey question	Description	% Yes	95% CI
Q1	Is your pet a dog?	90.5	86.0–93.9
Q1	Is your pet a cat?	9.5	6.1–14.0
Q2	Does your pet have regular access to children under the age of 18?	22.6	17.2–28.7
Q3	Where does your pet usually sleep? (Indoors in bed)	52.5	45.5–59.7
Q3	Where does your pet usually sleep? (Indoors not in bed)	47.1	39.8–53.8
Q3	Where does your pet usually sleep? (Outdoors)	0.5	0.0–1.4
Q4	Do you regularly (at least weekly) kiss your pet?	71.0	64.6–76.9
Q5	Does your pet regularly (at least weekly) lick you in the face?	57.5	50.7–64.1
Q6	Does your pet regularly (at least weekly) lick your hands?	78.3	72.3–83.5
Q7	Does your pet regularly (at least weekly) defecate inside your house, including in litterboxes or pads?	18.6	13.7–24.3
Q8	Has your pet taken an antibiotic by mouth or injection in the past 3 months?	38.5	32.0–45.2
Q9	Has your pet taken an antibiotic by mouth or injection in the past 12 months?	49.8	43.0–56.6
Q10	Have other pets in your household taken an antibiotic by mouth or injection in the past 12 months?	25.8	20.2–32.1
Q11	Has your pet been kenneled/boarded in the past 12 months?	34.8	28.6–41.5
Q12	Has your pet been hospitalized in the previous 3 months?	23.1	17.7–29.2
Q13	Has your pet been hospitalized in the previous 12 months?	27.1	21.4–33.5
Q14	Has your pet visited a human hospital in the past 12 months?	2.3	0.7–5.2
Q15	Has your pet visited a long-term care facility (nursing home) in the past 12 months?	1.8	0.5–4.6
Q16	Does your pet have regular access (at least weekly) to a stream, river, pond, or lake?	17.2	12.5–22.8
Q17	Does your pet regularly (at least weekly) consume water from the toilet?	6.8	3.8–10.9
Q18	Does your pet regularly (at least weekly) eat other animals’ feces?	9.0	5.6–13.6
Q19	Does your pet regularly (at least weekly) eat raw meat or a raw meat based diet?	1.8	0.5–4.6
Q20	Are you, or someone else in your household, employed in the human healthcare field or in a healthcare training program (direct patient care)?	14.0	9.7–19.3
Q21	Are you, or someone else in your household, employed in the veterinary field or in a veterinary training program (direct patient care)?	23.1	17.7–29.2
Q22	Have you, or someone else in your household, taken an antibiotic by mouth or injection in the past 3 months?	27.6	21.8–34.0
Q23	Have you, or someone else in your household, taken an antibiotic by mouth or injection in the past 12 months?	43.0	36.4–49.8
Q24	Have you, or someone else in your household, been hospitalized in the past 3 months?	4.1	1.9–7.6
Q25	Have you, or someone else in your household, been hospitalized in the past 12 months?	9.5	6.0–14.2
Q26	Have you, or someone else in your household, traveled internationally in the past 12 months?	24.0	18.5–30.2
Q27	Have any other pets or pet owners from your household participated in this study?	9.5	6.0–14.2

### Statistical analysis of survey variables and bacterial carriage

Univariate logistic regression was performed to explore associations between survey variables and bacterial carriage outcomes (Tables 3, 4). In humans (*n* = 221), evaluated outcomes included carriage of any Enterobacterales and *Klebsiella* spp. Employment in the veterinary field demonstrated a positive association with oral *Klebsiella* spp. carriage, including household veterinary employment (OR = 2.537, 95% CI: 0.963–6.688, *p* = 0.060). No biologically plausible positive associations with *p* < 0.10 were identified for carriage of any Enterobacterales in humans.

**Table 3:** Univariate logistic regression results for humans (OR > 1.0 and *p* ≤ 0.30).

Outcome	Variable	Description	OR	95% CI	*p*-value
Any Enterobacterales	Q21	Household member employed in veterinary field	1.543	0.679–3.508	0.300
Any Enterobacterales	Q26	Household member traveled internationally in past 12 months	1.833	0.819–4.105	0.141
*Klebsiella* spp.	Q21	Household member employed in veterinary field	2.537	0.963–6.688	0.060

**Table 4:** Univariate logistic regression results for dogs (OR > 1.0 and *p* ≤ 0.30).

Outcome	Variable	Description	OR	95% CI	p-value
Any Enterobacterales	Q8	Pet received antibiotic in past 3 months	1.565	0.849–2.887	0.151
Any Enterobacterales	Q9	Pet received antibiotic in past 12 months	1.786	0.973–3.276	0.061
Any Enterobacterales	Q10	Other pets received antibiotic in past 12 months	1.603	0.826–3.110	0.163
Any Enterobacterales	Q19	Pet regularly eats raw meat	3.380	0.345–33.154	0.296
Any Enterobacterales	Q22	Household member took antibiotic in past 3 months	1.526	0.777–2.995	0.220
Any Enterobacterales	Q25	Household member hospitalized in past 12 months	2.382	0.849–6.684	0.099
*Klebsiella* spp.	Q5	Pet regularly licks owner’s face	2.611	0.708–9.627	0.150
*Klebsiella* spp.	Q9	Pet received antibiotic in past 12 months	3.850	1.046–14.173	0.043
*Klebsiella* spp.	Q12	Pet hospitalized in past 3 months	4.134	1.400–12.213	0.010
*Klebsiella* spp.	Q13	Pet hospitalized in past 12 months	2.476	0.845–7.254	0.098
*Klebsiella* spp.	Q23	Household member took antibiotic in past 12 months	1.963	0.667–5.782	0.221
*E. coli*	Q4	Owner regularly kisses pet	1.791	0.831–3.859	0.137
*E. coli*	Q8	Pet received antibiotic in past 3 months	2.299	1.188–4.447	0.013
*E. coli*	Q9	Pet received antibiotic in past 12 months	3.049	1.517–6.129	0.002
*E. coli*	Q13	Pet hospitalized in past 12 months	1.734	0.861–3.493	0.123
*E. coli*	Q22	Household member took antibiotic in past 3 months	1.596	0.788–3.233	0.194
*E. coli*	Q23	Household member took antibiotic in past 12 months	1.600	0.834–3.069	0.157

In dogs (n = 197), evaluated outcomes included carriage of any Enterobacterales, *Klebsiella* spp., and *E. coli*. Prior antibiotic administration within the past 12 months demonstrated positive associations with multiple bacterial outcomes. Dogs receiving antibiotics within the previous 12 months had increased odds of *Klebsiella* spp. carriage (OR = 3.850, 95% CI: 1.046–14.173, *p* = 0.043) and *E. coli* carriage (OR = 3.049, 95% CI: 1.517–6.129, *p* = 0.002). Pet hospitalization within the previous 3 months was also positively associated with *Klebsiella* spp. carriage (OR = 4.134, 95% CI: 1.400–12.213, *p* = 0.010). A weaker positive association was observed between prior antibiotic exposure and carriage of any Enterobacterales (OR = 1.786, 95% CI: 0.973–3.276, *p* = 0.061).

### Whole-genome sequencing and genomic analysis

Whole-genome sequencing (WGS) was performed on 39 Enterobacterales phenotypically identified as *Klebsiella* spp. or *Enterobacter* spp. from human and canine samples (21 human isolates and 18 canine isolates), but three isolates failed pre-defined completeness or contamination thresholds, resulting in 36 isolates available for analysis (19 human isolates and 17 canine isolates).

Isolates were sequenced to an average estimated depth of 325.97X (SD 48.37X, Min 244.86X). Assemblies contained an average of 176.75 contigs (SD 98.23) and an average N50 of 129,164.72 bp (SD 44,537.28 bp). Per CheckM, included assemblies had an average of 98.05% completeness (SD 2.04%) and 1.48% contamination (SD 1.40%).

Sequencing data including host species, bacterial species, multilocus sequence type (MLST), and detected resistance genes are summarized in Figure 1. Isolates demonstrated substantial genomic diversity, with numerous unique sequence types identified across both human and canine samples. Only two repeated sequence types were identified among isolates. A previously unassigned *Klebsiella pneumoniae* sequence type was detected in one human and one canine isolate, sharing the same MLST allelic profile (gapA(18), infB(22), mdh(26), pgi(63), phoE(127), rpoB(20), tonB(51)); however, these isolates did not originate from a matched human-animal pair*. Klebsiella pneumoniae* ST20 was identified in two human isolates from separate households.

**Figure 1:**
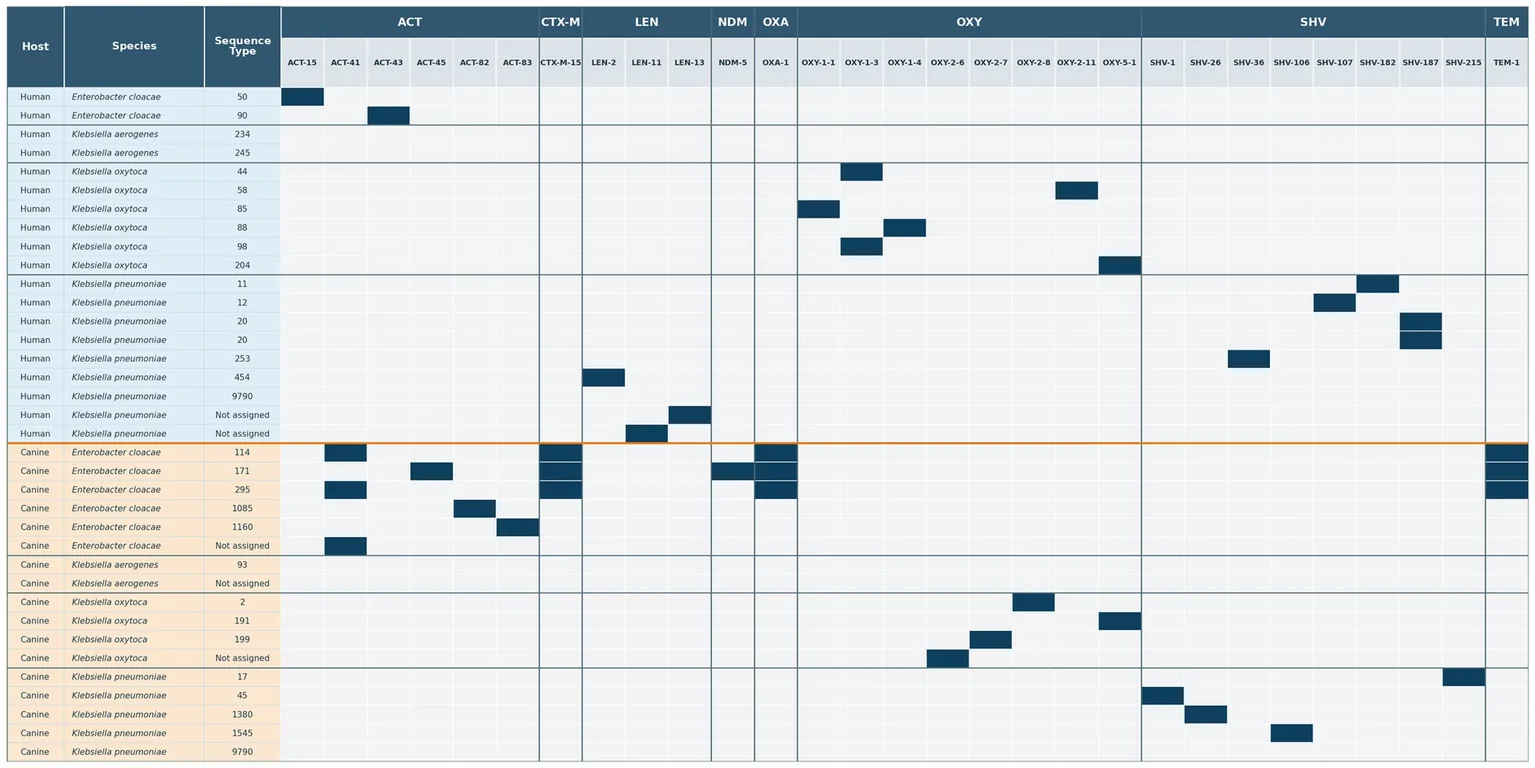
Isolate host, species, MLST sequence type assignment, and beta-lactamase gene heat map.

Resistance genes were commonly detected in both human and canine isolates. At least one beta-lactamase gene was identified in 84.2% (16/19) of human isolates and 82.4% (14/17) of canine isolates. Detected beta-lactamase families included ACT, CTX-M, LEN, NDM, OXA, OXY, SHV, and TEM. Notably, a canine *Enterobacter cloacae* isolate carried the carbapenemase gene NDM-5.

### Database uploads

The previously unassigned *Klebsiella pneumoniae* sequence type identified in a non-paired human and canine isolate was submitted to the Institut Pasteur Klebsiella MLST database and assigned sequence type ST9790. Sequencing data for all 39 isolates were deposited into the publicly available National Center for Biotechnology Information (NCBI) GenBank database under BioProject accession number PRJNA1467742.

## Discussion

This study explored survey-based risk factors associated with oral and oropharyngeal Enterobacterales carriage in humans and companion animals and characterized selected isolates using whole-genome sequencing within a One Health framework. In humans, employment in the veterinary field showed a nonsignificant positive trend toward oral *Klebsiella* spp. carriage, potentially reflecting increased occupational exposure to animals and veterinary healthcare environments. Similar occupational associations have been described in healthcare-associated antimicrobial resistance studies, including veterinary-specific investigations (40). No biologically plausible positive associations with *p* < 0.10 were identified for carriage of any Enterobacterales in humans, suggesting that additional unmeasured environmental, behavioral, or host-related factors may contribute to oral carriage dynamics.

Although Enterobacterales predominantly colonize the gastrointestinal tract, Gram-negative bacilli may be detected transiently in the oral cavity of healthy individuals. Potential routes of introduction include food, water, environmental exposure, and fecal–oral or hand-to-mouth transfer, while antimicrobial exposure, healthcare contact, oral health, and other host factors may influence persistence. The oral cavity may therefore serve as an intermittent reservoir even when gastrointestinal carriage is not assessed (30, 41).

In dogs, prior antibiotic exposure within the previous 12 months demonstrated positive associations with multiple bacterial outcomes, including *Klebsiella* spp. and *E. coli* carriage. These findings are consistent with previous studies demonstrating that antimicrobial exposure may increase Enterobacterales carriage and alter microbial ecology in companion animals (15). The observed association between antibiotic exposure and bacterial carriage supports concerns regarding antimicrobial selective pressure within veterinary patients and highlights the importance of antimicrobial stewardship in companion animal medicine.

Recent hospitalization was also positively associated with canine *Klebsiella* spp. carriage. This finding may reflect increased exposure to healthcare-associated bacterial populations, antimicrobial selection pressure, or environmental contamination within veterinary medical settings. Similar associations between hospitalization and multidrug-resistant Enterobacterales carriage have been well documented in human healthcare settings and may represent an important but underrecognized risk factor in companion animals (42).

Taken together, the clearest exploratory risk factors identified in this study were antimicrobial exposure and recent hospitalization in dogs. Veterinary occupational exposure in humans should be regarded as a hypothesis-generating signal rather than a statistically significant association.

Whole-genome sequencing identified substantial genomic diversity among isolates recovered from humans and dogs. The beta-lactamase profile spanned all four Ambler classes, including class A enzymes (CTX-M, SHV, TEM, OXY, and LEN), the class B metallo-beta-lactamase NDM-5, class C ACT-type enzymes, and class D OXA enzymes. This breadth reflected both species-associated chromosomal beta-lactamases and acquired resistance determinants across a taxonomically diverse isolate collection. Multiple clinically important resistance determinants were identified, including CTX-M-15 and NDM-5. The detection of a NDM-5-producing isolates in a canine *Enterobacter cloacae* isolate is notable given the clinical importance of carbapenemase-producing Enterobacterales in both human and veterinary medicine (5, 43). Although no matched human–animal pair shared the same bacterial species or MLST designation, the presence of similar resistance mechanisms across host species highlights the interconnected nature of antimicrobial resistance ecology within community settings.

A previously unassigned *Klebsiella pneumoniae* sequence type was identified in both a human and canine isolate from unrelated households and was subsequently assigned ST9790 through the Institut Pasteur *Klebsiella* MLST database. Of particular One Health relevance, both the human and canine *Klebsiella pneumoniae* ST9790 isolates were indistinguishable by cgMLST across 629 loci and were assigned to CG16865. Whole-genome comparison also yielded a near-zero Mash distance of 1.43 × 10^−5^, with 9,994 of 10,000 shared sketch hashes, indicating that the isolates were very closely related at the resolution afforded by these genomic measures. This Mash distance was substantially smaller than the 0.0036 observed between the next-closest isolate pair in the study, the two ST20 isolates. Although these genomic similarity findings are compatible with cross-species transmission or exposure to a shared source, a phylogenomic analysis with a larger collection of isolates of this novel strain is necessary to determine direct transmission and its direction.

These findings support the continued use of a One Health approach for investigating antimicrobial resistance at the human-animal interface. The identification of clinically important resistance genes in both humans and companion animals underscores the importance of antimicrobial stewardship, continued genomic surveillance, and further investigation into environmental and community-associated transmission pathways (1).

### Limitations

Several limitations should be considered when interpreting these findings. The cross-sectional study design limits causal inference between identified exposures and bacterial carriage outcomes. Sparse outcomes for several bacterial categories limited robust multivariable modeling and resulted in reliance on exploratory unadjusted logistic regression analyses. As a result, reported associations should be interpreted cautiously and considered hypothesis-generating. Because enrollment was not restricted to a single one-human–one-pet dyad, some observations arose from the same households; the exploratory analyses did not explicitly adjust for within-household clustering.

The study population was recruited from a veterinary tertiary referral center and included a relatively large proportion of participants employed in the veterinary field, which may limit generalizability to the broader human and companion animal population. Additionally, oral sampling was selected because of the known upper respiratory and oropharyngeal association of *Klebsiella* spp. and the practical advantages of non-invasive sample collection. However, oral sampling may underestimate overall Enterobacterales carriage compared to gastrointestinal sampling approaches (14, 27, 44). Accordingly, these findings describe oral and oropharyngeal carriage and should not be interpreted as estimates of overall colonization or as evidence that gastrointestinal carriage was absent. The use of an oral rinse in humans and targeted swabbing in animals may also have produced differential organism recovery, limiting direct prevalence comparisons between host species.

The feline sample was particularly limited: only 18 cats were enrolled and only one was culture-positive. This sample size is insufficient to estimate feline prevalence reliably or evaluate feline risk factors. A potentially lower frequency of feline oral/oropharyngeal Enterobacterales carriage may also have contributed to the low recovery, and the feline findings should not be generalized to the broader cat population.

Survey responses were self-reported and therefore subject to recall bias and potential misclassification. Responses of “Not sure” were grouped with negative responses to conservatively estimate risk, though this approach may have biased some associations toward the null. Whole-genome sequencing was limited to selected *Klebsiella* spp. and *Enterobacter* spp. isolates and therefore does not fully characterize the broader Enterobacterales population identified within the study. Future studies incorporating larger sample sizes, longitudinal sampling, gastrointestinal sampling strategies, and broader genomic characterization may provide additional insight into antimicrobial resistance ecology and transmission dynamics at the human-animal interface.

## Conclusion

This study identified exploratory associations between antimicrobial exposure and oral/oropharyngeal Enterobacterales carriage in companion animals and demonstrated the presence of clinically important antimicrobial resistance determinants in both human and canine isolates. Whole-genome sequencing revealed substantial genomic diversity and identified resistance genes of public health importance, including CTX-M-15 and NDM-5, though direct household strain sharing was not demonstrated within this cohort. These findings contribute to the growing body of literature examining antimicrobial resistance within shared human-animal environments and support continued One Health surveillance and antimicrobial stewardship efforts in both human and veterinary medicine (45).

## Data Availability

The datasets presented in this study can be found in online repositories. The names of the repository/repositories and accession number(s) can be found at: https://www.ncbi.nlm.nih.gov/genbank/, PRJNA1467742.
