# Supplementary material for: Survey-based risk factors and genomic characterization of oral and oropharyngeal Enterobacterales carriage in humans and companion animals: a One Health study
Source: Front Vet Sci. 2026 Sep 21;13:1900404. doi: 10.3389/fvets.2026.1900404 (PMC13634867; doi:10.3389/fvets.2026.1900404)
Supplement: Supplementary file 1 [file Table_1.docx]

Supplemental Table 1: Univariate Logistic Regression Results for Human vs Any Enterobacterales (n=221, 35 Positive)

| Outcome | Variable | Description | OR | P-value | 95% CI Lower | 95% CI Upper |
| --- | --- | --- | --- | --- | --- | --- |
| Any Enterobacterales | Q2 | Pet has regular access to children under 18 | 0.951 | 0.913 | 0.385 | 2.349 |
| Any Enterobacterales | Q3 | Pet usually sleeps indoors | 0.419 | 0.030 | 0.191 | 0.918 |
| Any Enterobacterales | Q4 | Owner regularly kisses pet | 0.540 | 0.119 | 0.249 | 1.173 |
| Any Enterobacterales | Q5 | Pet regularly licks owner’s face | 0.814 | 0.592 | 0.384 | 1.727 |
| Any Enterobacterales | Q6 | Pet regularly licks owner’s hands | 0.554 | 0.162 | 0.242 | 1.267 |
| Any Enterobacterales | Q7 | Pet regularly defecates indoors | 0.787 | 0.646 | 0.284 | 2.185 |
| Any Enterobacterales | Q8 | Pet received antibiotic in past 3 months | 0.691 | 0.366 | 0.310 | 1.541 |
| Any Enterobacterales | Q9 | Pet received antibiotic in past 12 months | 1.356 | 0.429 | 0.638 | 2.883 |
| Any Enterobacterales | Q10 | Other pets received antibiotic in past 12 months | 0.952 | 0.912 | 0.401 | 2.259 |
| Any Enterobacterales | Q11 | Pet kenneled/boarded in past 12 months | 0.976 | 0.952 | 0.444 | 2.148 |
| Any Enterobacterales | Q12 | Pet hospitalized in past 3 months | 0.738 | 0.531 | 0.286 | 1.907 |
| Any Enterobacterales | Q13 | Pet hospitalized in past 12 months | 0.878 | 0.768 | 0.371 | 2.078 |
| Any Enterobacterales | Q14 | Pet visited human hospital in past 12 months | 0.000 | 0.999 | 0.000 | - |
| Any Enterobacterales | Q15 | Pet visited long-term care facility in past 12 months | 0.000 | 0.999 | 0.000 | - |
| Any Enterobacterales | Q16 | Pet has regular access to stream/river/pond/lake | 0.875 | 0.799 | 0.314 | 2.441 |
| Any Enterobacterales | Q17 | Pet regularly consumes toilet water | 0.903 | 0.896 | 0.194 | 4.203 |
| Any Enterobacterales | Q18 | Pet regularly eats other animals’ feces | 0.633 | 0.554 | 0.140 | 2.871 |
| Any Enterobacterales | Q19 | Pet regularly eats raw meat | 0.000 | 0.999 | 0.000 | - |
| Any Enterobacterales | Q20 | Household member employed in human healthcare | 0.368 | 0.187 | 0.083 | 1.624 |
| Any Enterobacterales | Q21 | Household member employed in veterinary field | 1.543 | 0.300 | 0.679 | 3.508 |
| Any Enterobacterales | Q22 | Household member took antibiotic in past 3 months | 1.031 | 0.943 | 0.448 | 2.374 |
| Any Enterobacterales | Q23 | Household member took antibiotic in past 12 months | 0.556 | 0.151 | 0.250 | 1.238 |
| Any Enterobacterales | Q24 | Household member hospitalized in past 3 months | 0.730 | 0.770 | 0.088 | 6.041 |
| Any Enterobacterales | Q25 | Household member hospitalized in past 12 months | 0.000 | 0.998 | 0.000 | - |
| Any Enterobacterales | Q26 | Household member traveled internationally in past 12 months | 1.833 | 0.141 | 0.819 | 4.105 |

Supplemental Table 2: Univariate Logistic Regression Results for Human vs Klebsiella spp. (n=221, 19 Positive)

| Outcome | Variable | Description | OR | P-value | 95% CI Lower | 95% CI Upper |
| --- | --- | --- | --- | --- | --- | --- |
| *Klebsiella* spp. | Q2 | Pet has regular access to children under 18 | 0.618 | 0.460 | 0.173 | 2.214 |
| *Klebsiella* spp. | Q3 | Pet usually sleeps indoors | 0.386 | 0.064 | 0.141 | 1.056 |
| *Klebsiella* spp. | Q4 | Owner regularly kisses pet | 0.873 | 0.792 | 0.317 | 2.406 |
| *Klebsiella* spp. | Q5 | Pet regularly licks owner’s face | 1.297 | 0.600 | 0.490 | 3.431 |
| *Klebsiella* spp. | Q6 | Pet regularly licks owner’s hands | 0.569 | 0.281 | 0.204 | 1.586 |
| *Klebsiella* spp. | Q7 | Pet regularly defecates indoors | 1.189 | 0.770 | 0.373 | 3.790 |
| *Klebsiella* spp. | Q8 | Pet received antibiotic in past 3 months | 0.719 | 0.520 | 0.262 | 1.969 |
| *Klebsiella* spp. | Q9 | Pet received antibiotic in past 12 months | 1.133 | 0.795 | 0.442 | 2.907 |
| *Klebsiella* spp. | Q10 | Other pets received antibiotic in past 12 months | 0.750 | 0.622 | 0.238 | 2.360 |
| *Klebsiella* spp. | Q11 | Pet kenneled/boarded in past 12 months | 1.100 | 0.848 | 0.414 | 2.920 |
| *Klebsiella* spp. | Q12 | Pet hospitalized in past 3 months | 1.211 | 0.726 | 0.414 | 3.541 |
| *Klebsiella* spp. | Q13 | Pet hospitalized in past 12 months | 1.640 | 0.324 | 0.613 | 4.385 |
| *Klebsiella* spp. | Q14 | Pet visited human hospital in past 12 months | 0.000 | 0.999 | 0.000 | - |
| *Klebsiella* spp. | Q15 | Pet visited long-term care facility in past 12 months | 0.000 | 0.999 | 0.000 | - |
| *Klebsiella* spp. | Q16 | Pet has regular access to stream/river/pond/lake | 0.895 | 0.865 | 0.247 | 3.236 |
| *Klebsiella* spp. | Q17 | Pet regularly consumes toilet water | 0.746 | 0.783 | 0.093 | 6.005 |
| *Klebsiella* spp. | Q18 | Pet regularly eats other animals’ feces | 0.535 | 0.553 | 0.068 | 4.233 |
| *Klebsiella* spp. | Q19 | Pet regularly eats raw meat | 0.000 | 0.999 | 0.000 | - |
| *Klebsiella* spp. | Q20 | Household member employed in human healthcare | 0.702 | 0.647 | 0.154 | 3.199 |
| *Klebsiella* spp. | Q21 | Household member employed in veterinary field | 2.537 | 0.060 | 0.963 | 6.688 |
| *Klebsiella* spp. | Q22 | Household member took antibiotic in past 3 months | 0.678 | 0.506 | 0.216 | 2.131 |
| *Klebsiella* spp. | Q23 | Household member took antibiotic in past 12 months | 0.444 | 0.133 | 0.154 | 1.280 |
| *Klebsiella* spp. | Q24 | Household member hospitalized in past 3 months | 0.000 | 0.999 | 0.000 | - |
| *Klebsiella* spp. | Q25 | Household member hospitalized in past 12 months | 0.000 | 0.998 | 0.000 | - |
| *Klebsiella* spp. | Q26 | Household member traveled internationally in past 12 months | 1.146 | 0.803 | 0.393 | 3.345 |

Supplemental Table 3: Univariate Logistic Regression Results for Canine vs Any Enterobacterales (n=197, 93 Positive)

| Outcome | Variable | Description | OR | P-value | 95% CI Lower | 95% CI Upper |
| --- | --- | --- | --- | --- | --- | --- |
| Any Enterobacterales | Q2 | Pet has regular access to children under 18 | 0.762 | 0.455 | 0.373 | 1.555 |
| Any Enterobacterales | Q3 | Pet usually sleeps indoors | 0.930 | 0.814 | 0.509 | 1.699 |
| Any Enterobacterales | Q4 | Owner regularly kisses pet | 0.739 | 0.373 | 0.381 | 1.436 |
| Any Enterobacterales | Q5 | Pet regularly licks owner’s face | 0.905 | 0.750 | 0.488 | 1.677 |
| Any Enterobacterales | Q6 | Pet regularly licks owner’s hands | 1.236 | 0.601 | 0.559 | 2.733 |
| Any Enterobacterales | Q7 | Pet regularly defecates indoors | 1.110 | 0.835 | 0.418 | 2.946 |
| Any Enterobacterales | Q8 | Pet received antibiotic in past 3 months | 1.565 | 0.151 | 0.849 | 2.887 |
| Any Enterobacterales | Q9 | Pet received antibiotic in past 12 months | 1.786 | 0.061 | 0.973 | 3.276 |
| Any Enterobacterales | Q10 | Other pets received antibiotic in past 12 months | 1.603 | 0.163 | 0.826 | 3.110 |
| Any Enterobacterales | Q11 | Pet kenneled/boarded in past 12 months | 0.987 | 0.967 | 0.525 | 1.854 |
| Any Enterobacterales | Q12 | Pet hospitalized in past 3 months | 0.997 | 0.993 | 0.497 | 2.001 |
| Any Enterobacterales | Q13 | Pet hospitalized in past 12 months | 1.014 | 0.968 | 0.520 | 1.975 |
| Any Enterobacterales | Q14 | Pet visited human hospital in past 12 months | 0.265 | 0.240 | 0.029 | 2.425 |
| Any Enterobacterales | Q15 | Pet visited long-term care facility in past 12 months | 2.225 | 0.517 | 0.198 | 25.006 |
| Any Enterobacterales | Q16 | Pet has regular access to stream/river/pond/lake | 0.977 | 0.951 | 0.468 | 2.040 |
| Any Enterobacterales | Q17 | Pet regularly consumes toilet water | 1.307 | 0.644 | 0.420 | 4.061 |
| Any Enterobacterales | Q18 | Pet regularly eats other animals’ feces | 1.107 | 0.856 | 0.371 | 3.302 |
| Any Enterobacterales | Q19 | Pet regularly eats raw meat | 3.380 | 0.296 | 0.345 | 33.154 |
| Any Enterobacterales | Q20 | Household member employed in human healthcare | 0.793 | 0.578 | 0.350 | 1.794 |
| Any Enterobacterales | Q21 | Household member employed in veterinary field | 0.667 | 0.269 | 0.325 | 1.368 |
| Any Enterobacterales | Q22 | Household member took antibiotic in past 3 months | 1.526 | 0.220 | 0.777 | 2.995 |
| Any Enterobacterales | Q23 | Household member took antibiotic in past 12 months | 1.241 | 0.482 | 0.680 | 2.267 |
| Any Enterobacterales | Q24 | Household member hospitalized in past 3 months | 1.103 | 0.893 | 0.267 | 4.559 |
| Any Enterobacterales | Q25 | Household member hospitalized in past 12 months | 2.382 | 0.099 | 0.849 | 6.684 |
| Any Enterobacterales | Q26 | Household member traveled internationally in past 12 months | 1.393 | 0.356 | 0.689 | 2.818 |

Supplemental Table 4: Univariate Logistic Regression Results for Canine vs Klebsiella spp. (n=197, 16 Positive)

| Outcome | Variable | Description | OR | P-value | 95% CI Lower | 95% CI Upper |
| --- | --- | --- | --- | --- | --- | --- |
| *Klebsiella* spp. | Q2 | Pet has regular access to children under 18 | 1.222 | 0.744 | 0.367 | 4.072 |
| *Klebsiella* spp. | Q3 | Pet usually sleeps indoors | 1.207 | 0.733 | 0.410 | 3.553 |
| *Klebsiella* spp. | Q4 | Owner regularly kisses pet | 0.566 | 0.306 | 0.190 | 1.684 |
| *Klebsiella* spp. | Q5 | Pet regularly licks owner’s face | 2.611 | 0.150 | 0.708 | 9.627 |
| *Klebsiella* spp. | Q6 | Pet regularly licks owner’s hands | 1.411 | 0.662 | 0.301 | 6.606 |
| *Klebsiella* spp. | Q7 | Pet regularly defecates indoors | 1.356 | 0.705 | 0.280 | 6.555 |
| *Klebsiella* spp. | Q8 | Pet received antibiotic in past 3 months | 1.751 | 0.302 | 0.604 | 5.073 |
| *Klebsiella* spp. | Q9 | Pet received antibiotic in past 12 months | 3.850 | 0.043 | 1.046 | 14.173 |
| *Klebsiella* spp. | Q10 | Other pets received antibiotic in past 12 months | 0.585 | 0.423 | 0.158 | 2.169 |
| *Klebsiella* spp. | Q11 | Pet kenneled/boarded in past 12 months | 0.451 | 0.232 | 0.122 | 1.665 |
| *Klebsiella* spp. | Q12 | Pet hospitalized in past 3 months | 4.134 | 0.010 | 1.400 | 12.213 |
| *Klebsiella* spp. | Q13 | Pet hospitalized in past 12 months | 2.476 | 0.098 | 0.845 | 7.254 |
| *Klebsiella* spp. | Q14 | Pet visited human hospital in past 12 months | 2.732 | 0.383 | 0.285 | 26.146 |
| *Klebsiella* spp. | Q15 | Pet visited long-term care facility in past 12 months | 0.000 | 0.999 | 0.000 | - |
| *Klebsiella* spp. | Q16 | Pet has regular access to stream/river/pond/lake | 0.939 | 0.926 | 0.250 | 3.524 |
| *Klebsiella* spp. | Q17 | Pet regularly consumes toilet water | 2.042 | 0.385 | 0.408 | 10.214 |
| *Klebsiella* spp. | Q18 | Pet regularly eats other animals’ feces | 1.859 | 0.448 | 0.375 | 9.216 |
| *Klebsiella* spp. | Q19 | Pet regularly eats raw meat | 0.000 | 0.999 | 0.000 | - |
| *Klebsiella* spp. | Q20 | Household member employed in human healthcare | 0.775 | 0.747 | 0.165 | 3.641 |
| *Klebsiella* spp. | Q21 | Household member employed in veterinary field | 0.216 | 0.145 | 0.028 | 1.697 |
| *Klebsiella* spp. | Q22 | Household member took antibiotic in past 3 months | 1.369 | 0.586 | 0.442 | 4.239 |
| *Klebsiella* spp. | Q23 | Household member took antibiotic in past 12 months | 1.963 | 0.221 | 0.667 | 5.782 |
| *Klebsiella* spp. | Q24 | Household member hospitalized in past 3 months | 0.000 | 0.999 | 0.000 | - |
| *Klebsiella* spp. | Q25 | Household member hospitalized in past 12 months | 0.615 | 0.650 | 0.076 | 5.003 |
| *Klebsiella* spp. | Q26 | Household member traveled internationally in past 12 months | 0.207 | 0.134 | 0.026 | 1.626 |

Supplemental Table 5: Univariate Logistic Regression Results for Canine vs E. coli. (n=197, 60 Positive)

| Outcome | Variable | Description | OR | P-value | 95% CI Lower | 95% CI Upper |
| --- | --- | --- | --- | --- | --- | --- |
| *E. coli* | Q2 | Pet has regular access to children under 18 | 0.951 | 0.899 | 0.440 | 2.055 |
| *E. coli* | Q3 | Pet usually sleeps indoors | 1.171 | 0.637 | 0.608 | 2.253 |
| *E. coli* | Q4 | Owner regularly kisses pet | 1.791 | 0.137 | 0.831 | 3.859 |
| *E. coli* | Q5 | Pet regularly licks owner’s face | 0.894 | 0.741 | 0.460 | 1.737 |
| *E. coli* | Q6 | Pet regularly licks owner’s hands | 1.276 | 0.589 | 0.528 | 3.085 |
| *E. coli* | Q7 | Pet regularly defecates indoors | 1.138 | 0.807 | 0.403 | 3.215 |
| *E. coli* | Q8 | Pet received antibiotic in past 3 months | 2.299 | 0.013 | 1.188 | 4.447 |
| *E. coli* | Q9 | Pet received antibiotic in past 12 months | 3.049 | 0.002 | 1.517 | 6.129 |
| *E. coli* | Q10 | Other pets received antibiotic in past 12 months | 1.398 | 0.347 | 0.696 | 2.810 |
| *E. coli* | Q11 | Pet kenneled/boarded in past 12 months | 0.978 | 0.950 | 0.494 | 1.937 |
| *E. coli* | Q12 | Pet hospitalized in past 3 months | 1.167 | 0.684 | 0.555 | 2.453 |
| *E. coli* | Q13 | Pet hospitalized in past 12 months | 1.734 | 0.123 | 0.861 | 3.493 |
| *E. coli* | Q14 | Pet visited human hospital in past 12 months | 0.000 | 0.999 | 0.000 | - |
| *E. coli* | Q15 | Pet visited long-term care facility in past 12 months | 1.125 | 0.924 | 0.100 | 12.684 |
| *E. coli* | Q16 | Pet has regular access to stream/river/pond/lake | 1.159 | 0.713 | 0.529 | 2.537 |
| *E. coli* | Q17 | Pet regularly consumes toilet water | 0.654 | 0.532 | 0.172 | 2.480 |
| *E. coli* | Q18 | Pet regularly eats other animals’ feces | 0.890 | 0.850 | 0.266 | 2.977 |
| *E. coli* | Q19 | Pet regularly eats raw meat | 0.744 | 0.800 | 0.076 | 7.319 |
| *E. coli* | Q20 | Household member employed in human healthcare | 1.077 | 0.868 | 0.452 | 2.566 |
| *E. coli* | Q21 | Household member employed in veterinary field | 0.483 | 0.095 | 0.206 | 1.136 |
| *E. coli* | Q22 | Household member took antibiotic in past 3 months | 1.596 | 0.194 | 0.788 | 3.233 |
| *E. coli* | Q23 | Household member took antibiotic in past 12 months | 1.600 | 0.157 | 0.834 | 3.069 |
| *E. coli* | Q24 | Household member hospitalized in past 3 months | 1.368 | 0.676 | 0.315 | 5.947 |
| *E. coli* | Q25 | Household member hospitalized in past 12 months | 1.519 | 0.418 | 0.553 | 4.173 |
| *E. coli* | Q26 | Household member traveled internationally in past 12 months | 1.450 | 0.326 | 0.691 | 3.042 |
